# Linking type 2 diabetes mellitus, cardiac hypertrophy and depression in a diurnal animal model

**DOI:** 10.1038/s41598-019-48326-7

**Published:** 2019-08-14

**Authors:** Carmel Bilu, Haim Einat, Orly Barak, Paul Zimmet, Vicktoria Vishnevskia-Dai, Amanda Govrin, Galila Agam, Noga Kronfeld-Schor

**Affiliations:** 1 School of Zoology, Tel-Aviv University, Tel Aviv, Ramat Aviv Israel; 2 Department of Clinical Biochemistry and Pharmacology, Ben-Gurion University of the Negev, Beer Sheva, Israel; 3 School of Behavioral Sciences, Tel Aviv-Yaffo Academic College, Tel-Aviv, Israel; 4 Department of Diabetes, Central Clinical School, Monash University, Melbourne, Australia; 5 Ocular Oncology and Autoimmune service, The Goldschleger Eye Institute, The Chaim Sheba Medical Center, Tel-Hashomer, Sackler Faculty of Medicine, Tel-Aviv University, Tel Aviv, Israel

**Keywords:** Animal physiology, Metabolic syndrome

## Abstract

It was recently suggested that the Metabolic Syndrome should be renamed to “Circadian Syndrome”. In this context, we explored the effects of living under standard laboratory conditions, where light is the only cycling variable (relevant to human modern life), in a diurnal mammal, on the relationships between affective-like pathology, type 2 diabetes mellitus (T2DM), and cardiac hypertrophy. After 20 weeks, some of the animals spontaneously developed T2DM, depressive and anxiety-like behavior and cardiac hypertrophy. There were significant correlations between levels of anxiety-like behavior and glucose tolerance, and between heart/total body weight ratio and glucose tolerance. Our data suggest a relationship between the development of T2DM, emotional and cardiac pathology as seen in diurnal humans. Furthermore, our data show a possible relationship between reduced daily cycling cues in the laboratory and what has been regularly termed “Metabolic Syndrome” and recently proposed by us to be renamed to “Circadian Syndrome”.

## Introduction

The comorbid relationship between depression, cardiovascular diseases (CVD) and type 2 diabetes mellitus (T2DM) is repeatedly described in the literature^1–7^. Whereas the underlying mechanisms connecting CVD and T2DM are somewhat understood^3,8^, the relationship between these two very common chronic diseases with depression is well-documented clinically but not yet understood at the mechanistic level^1,9–15^.

Yet, depression^16–18^, CVD^19–24^ and T2DM^25,26^ have been strongly linked with circadian rhythm disturbances. Moreover, it was recently suggested that circadian disruption might be an important underlying and uniting etiological factor for the key cardio-metabolic components of the Metabolic Syndrome and its associated comorbidities: sleep disturbances, depression, steatohepatitis and cognitive dysfunction. We have suggested that this combination of the Metabolic Syndrome and the comorbidities warrants that it should be renamed the “Circadian Syndrome”^15^.

The fat sand rat (*Psammomys obesus*) is a large burrow-dwelling gerbil (160 ± 30 g) that inhabits wadi beds, saline and saline-marsh plains in the deserts of North Africa, from Mauritania to Egypt, Sudan and Israel^27^. Studies from the 1960’s discovered that when sand rats are held under laboratory conditions and fed standard rodent food they quickly develop diabetes^28,29^. As a result, it has become a frequently used animal model to explore the underlying biology of T2DM^30–34^.

Sand rats in nature are strictly diurnal, spending extended periods in foraging on salt bushes^35^. When brought into laboratory conditions, they demonstrate an unstable, nocturnal phase preference, with low amplitude and, in some cases, no rhythm at all^36,37^. A switch from clear diurnality in nature to a mixed diurnal/nocturnal pattern in the laboratory is not uncommon and has been described for the golden spiny mouse (*Acomys russatus*)^38^, Nile grass rat (*Arvicanthis niloticus*)^39^, degu (*Octodon degu*)^40^, tuco-tuco (*Ctenomys aff*. *knighti*)^41^ and Mongolian gerbil (*Meriones unguiculatus*)^42^. No such responses have been reported in nocturnal rodents^36^, albeit mice with targeted mutation in the Per2 clock gene were shown to have less robust circadian rhythms when placed in a semi-natural environment^43^.

We recently demonstrated that relatively mild interference with circadian rhythms in the sand rat can accelerate the development of T2DM, obesity and cardiac hypertrophy^37^, and suggested that the possible underlying mechanism of this process is related to changes that accompany the switch from the mammalian ancestral nocturnal activity to the current diurnal one^37,44^. Interference with the sand rat circadian rhythms can also lead to the development of a depressive- and anxiety-like behavioral phenotype^45–47^ that is ameliorated by antidepressant treatment^48^, increased physical activity (voluntary wheel running)^49^ and bright white or blue light treatment^50–52^. Interestingly, similar circadian interventions in nocturnal mice did not result in depressive-like phenotypes^53^.

All things considered, data have shown that (1) when transferred into laboratory conditions most, but not all, sand rats develop T2DM. (2) Relatively small circadian manipulation induce a depression/anxiety-like phenotype in the sand rats. (3) Small manipulation of circadian rhythms in the sand rat accelerate the development of T2DM and cardiac hypertrophy. Based on these data we hypothesized that circadian disruption could be the underlying common denominator for the development of T2DM, obesity, CVD and depression and for the comorbidity between these four pathological states as a part of the newly termed “Circadian Syndrome”^15^.

To further explore this hypothesis, the current study examined correlations between the development of diabetes and depression/anxiety-like phenotype in sand rats maintained in laboratory settings under 12:12 LD conditions and fed standard rodent food.

## Results

### Development of T2DM

From 60 sand rats, 15 died during the 20-week period of eating standard rodent food. At the time of the glucose tolerance test, 13 out of the remaining 45 animals had glucose levels above 110 mg/dl and were considered diabetic and 32 had glucose levels lower than 110 mg/dl and were considered non-diabetic. Of the non-diabetic animals, we randomly selected 13 individuals for the experiment for further analyses to match the sample size of the diabetic group.

### Behavioral tests

To obtain an overall analysis of the difference between diabetic and non-diabetic animals in the behavioral tests we analyzed in one matrix one measure from each test: Sink 2 in the FST and time in the open arms in the EPM^54^. The analysis indicated a significant difference between the groups [ANOVA: F(2,22) = 4.5, p = 0.023]. We then analyzed separately the data for each test.

#### Forced swim test

**Figure 1:**
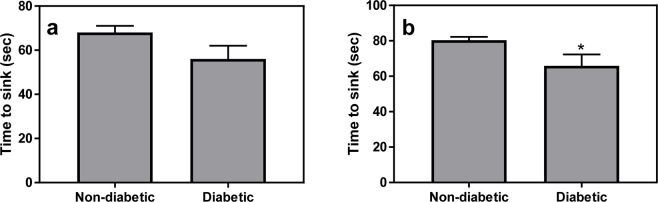
In the modified FST, diabetic fat sand rats exhibited a trend for lower time to Sink 1 (**a**) and a significantly lower time to sink 2 (**b**) compared with the non-diabetic animals. *Signifies p < 0.05 between the groups. N = 12 for non-diabetic group and 13 for diabetic group.

repeated measures ANOVA across Sink 1 and Sink 2 showed a significant “Sink” effect [F(1,23) = 63, p < 0.001, a near significant Diabetes effect [F(1,23) = 3.8, p = 0.063] and no interaction [F1,23) = 0.96, p = 0.34]. Following the significant “Sink” effect, we separately analyzed the Sink 1 and Sink 2 data. Data show a trend for difference for Sink 1 [Fig. 1a, t(23) = 1.7, p = 0.1] and a significant difference between diabetic and non-diabetic sand rats for Sink 2 [Fig. 1b, t(23) = 2.1, p = 0.047]. However, we found no correlation between sink time in the FST and glucose levels in the glucose tolerance test (data not shown, r = 0.26, p = 0.2).

#### Elevated plus maze

**Figure 2:**
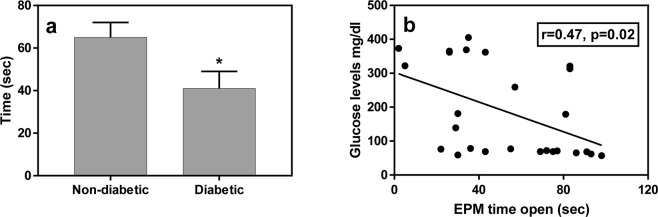
Diabetic fat sand rats spent less time in the open arms of the EPM than the non-diabetic animals (a, N = 13/group). The response in the EPM shows correlation with glucose levels in the GTT (b, N = 26). *Signifies p < 0.05 for the difference between the groups.

analysis of “time in open arms” indicated a significantly lower time in the open arms in the diabetic compared with non-diabetic animals [Fig. 2a; t (24) = 2.32, p = 0.03; effect size – Cohen’s d = 0.91]. A similar trend that did not reach statistical significance was demonstrated for the open/closed arms time ratio [t (24) = 2.01, p = 0.06]. Additionally, we found a significant correlation between the time in open arms and glucose levels in the glucose tolerance test for all animals combined (Fig. 2b; r = 0.47, p = 0.02).

### Weight and heart weight

**Figure 3:**
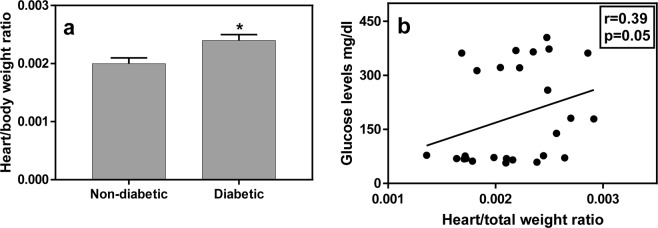
Diabetic animals exhibited a higher heart/total body weight ratio than the non-diabetic ones (a, N = 13/group). There was a significant correlation between the heart/total body weight ratio and glucose levels in the GTT (b, N = 26). *Signifies p < 0.05 for the difference between the groups.

Heart weights were larger in the diabetic groups [t (24) = 3.7, p = 0.001; effect size – Cohen’s d = 1.41] and similar results were demonstrated in the measure of heart/total body weight ratio [Fig. 3a; t (24) = 2.7, p = 0.013; effect size – Cohen’s d = 1.0]. No difference was detected in total body weight between non-diabetic and diabetic animals [t (24) = 1.0, p = 0.35]. Similar to the finding for the EPM, there was also a significant correlation between heart/total body weight ratio and glucose levels for all animals combined (Fig. 3b; r = 0.39, p = 0.05).

## Discussion

Studies from the 1960’s onwards show that when sand rats are brought into laboratory conditions and fed standard laboratory food, most, but not all, develop T2DM^28,29^. In the past, it was believed by most that this was due to the change in their diet in the laboratory^28,29,55^. It comes as a surprise that recent data show that when held outdoors in laboratory cages and fed standard rodent food sand rats do not develop T2DM, and that small circadian manipulation can result in depressive- and anxiety-like phenotype^47^ and in accelerated development of T2DM, obesity and cardiac hypertrophy^37^. With that in mind, the current study was designed to explore possible relationship between living under standard laboratory conditions, where light is the only cycling variable (relevant to human modern life), and the development of T2DM, CVD and depressive/anxiety-like phenotype in a diurnal mammal - the fat sand rat.

Indeed, the results replicate previous findings showing that HsdHu diabetes-prone sand rats develop T2DM under laboratory conditions. In the present experiment, approximately 50% of animals developed T2DM, whereas some of the previous studies reported higher rates at around 70%^32,56,57^. Interestingly, the animals developing T2DM show more depressive- and anxiety-like behavior compared with sand rats that did not develop diabetes. Moreover, animals that develop diabetes show cardiac hypertrophy. Together, the findings suggest a relationship between the development of diabetes, emotional pathology and cardiac pathology as seen in T2DM in human subjects^1,9–15^. Furthermore, that data raise a possible relationship between reduced daily cycling cues under laboratory conditions and what has regularly been termed the Metabolic Syndrome^15,58–61^, recently suggested to be termed the “Circadian Syndrome”^15^.

In humans, even small desynchronization between the internal clock and external light/dark cycle has been demonstrated to be linked to decreased metabolic efficiency and disrupted cardiac function^62^ as well as influence insulin secretion and metabolism, contributing to the development of insulin resistance^63^. Evidence shows that disturbed circadian rhythms could be implicated in key features of the Metabolic Syndrome as well as in sleep disturbances, depression, steatohepatitis and cognitive dysfunction forming the “Circadian Syndrome”^15^. The current and previous findings in the sand rats^64^ highlight disrupted or reduced circadian rhythms as a potential common denominator of these pathologies and suggest that the diurnal fat sand rat could be an advantageous animal model to further explore these relationships.

Sand rats in their natural environment do not develop T2DM, CVD, obesity or depressive-like behavior, but when exposed to small circadian interference pathologies appear^37,47^. Whereas in previous studies we used short photoperiod conditions as a circadian stressor, the current study explored the effects of standard laboratory conditions alone with no additional interventions. The rational for using laboratory conditions was twofold: (1) It is well-documented that sand rats kept in laboratory conditions lose or decrease their diurnal rhythms^65,66^. (2) It has been reported that many sand rats maintained in laboratory conditions develop T2DM^28,55,57,67^. In the laboratory the only cycling environmental condition is light, while under natural conditions a wealth of factors, both a-biotic (temperature, humidity, radiation) and biotic (competitors, predators, conspecifics) show a daily rhythm that may promote daytime activity^65^. This was also shown in laboratory conditions with mice where experimental food scarcity resulted in a switch to diurnal patterns and changes in energy balance^68^. Moreover, our standard laboratory light illuminance is around 800 lux, and wavelength is with a constant range of 420–780 nm^52^, while the range of natural light illuminance in Israel (the most northern range of sand rats) is between 7,500 and 80,000 lux (depending on season and cloud overcast)^69^ and light wavelength range between 300–2400 nm^70^. It is possible that the laboratory conditions are not sufficient for synchronizing the circadian system and promoting diurnal activity pattern^65^.

Laboratory conditions are similar in many ways to the modern Western lifestyle and living conditions: controlled ambient temperature and constant food availability^71^, low physical activity, no interspecific interactions, extensive use of artificial light during the night (“light pollution”) and low light exposure during the day^72–74^. Consequently, circadian rhythm disturbances were offered to be important contributors to the modern-day epidemics of T2DM, CVD and obesity^19,23,63,72,75–81^ through what we have recently suggested to be termed the “Circadian Syndrome“^15^. Recognizing the link between modern lifestyle and circadian disruption, risk and etiology of T2DM, CVD, obesity and depression that form the “Circadian Syndrome”, may have key implications for non-pharmacological prevention and therapeutic strategies to manage the contemporary and escalating non-communicable disease epidemic.

## Methods

### Animals

Sixty HsdHu diabetes-prone male fat sand rats (*Psammomys obesus*, 6–7 months old, from our colony at Tel Aviv University Zoological Research Garden) were used as subjects. As previously done^37^, animals were individually housed in standard plastic cages (42 cm × 26 cm × 15 cm) positioned in temperature-controlled rooms (25 °C) 12 hr/12 hr light/dark cycle with lights on at 08:00 and off at 20:00. Light intensity was 800 lux. Animals were provided *ad-lib* tap water and standard rodent food (product 19510; Koffolk, Petach-Tikva, Israel). All experimental procedures followed the NIH guidelines for the care and use of laboratory animals and were approved by the Institutional Animal Care and Use Committee (IACUC) of Tel Aviv University (permit number L15055).

### Procedure

Following former work from our laboratory^37^, animals were maintained in the colony conditions for 20 weeks before the start of any manipulation or testing. On week 20, Animals were weighed, blood glucose levels were measured, and oral glucose tolerance tests were performed. Behavioral tests for anxiety- and depressive- like behavior were conducted on week 21. On week 22, the sand rats were euthanized, the chest cavity was rapidly opened, the heart removed and weighed, and the heart weight/body weight ratio calculated.

### Glucose tolerance test (GTT)

Glucose tolerance tests were performed in animals fasted for 4 hours. Tests were performed at ZT 2 (ZT = Zeitgaber Time; ZT 0 = the time of lights-on) by administering 2 g glucose/kg body weight using gastric gavages (a syringe attached to a 20-gauge × 1.5 feeding needle), inserted through the mouth into the stomach^37^. When blood glucose values exceeded 110 mg/dl animals were considered as hyperglycemic^32^. We selected this relatively strict cutoff criterion suggested by Marquié and colleagues^32^ to allow as many animals as possible in the study. Based on the results the sand rats were divided into two groups – diabetic and healthy.

### Behavioral tests

On week 21 animals were evaluated in two standard behavioral tests of depression- and anxiety-like behaviors. The tests were performed in sequence, the Elevated Plus Maze (EPM) and the Forced Swim Test (FST).

#### Elevated Plus-Maze (EPM)

As explained in multiple previous work (e.g.^82^), the test presents the rodent with a conflict between its tendency to remain in a safe enclosed area and the need to explore new environments^83^. For the present study, we followed our standard procedure^84,85^. The maze was constructed from black aluminum and had two open arms (50 cm long and 10 cm wide) and two closed arms (same dimensions with 15 cm high walls). The plus maze was elevated 50 cm above the floor and light levels at the open arms were 200 lux. The test started an hour after light onset in the rooms (09:00) and animals were tested only during the next 3.5 hours. Order of testing was random within group with alternation between diabetic and non-diabetic animals. Sand rats were individually placed in the center of the maze and their behavior digitally recorded for a 5 min session. Recordings were used for later manual scoring of behaviors. At the end of the session, animals were returned to their cages and the maze was wiped clean with 70% ethanol before the start of the next session. Scoring included the time and the number of entries into each arm and was done by an investigator blind to treatment^84^.

#### Forced swim test

The FST is a commonly used test for the evaluation of depression-like behavior and assessment of antidepressants effects. As described in our previous papers^84,85^, the FST was used with several methodological alterations in sand rats [for review see^47^]. In the present study, we followed the established sand rats’ protocol as previously described^37,45,46^. Each animal was subjected to the FST twice over two consecutive days with the second exposure serving as the test session. Testing started an hour after the onset of lights and ended within the light period in the colony rooms. Each animal was placed individually into a white opaque cylinder, 30 cm in diameter and 45 cm high, filled with water (22–23 °C) to a depth of 25 cm. The test was digitally recorded from above for later manual scoring of behavior. As noted in previous work with sand rats, their ability to float is lower compared with rats or mice. Therefore, the standard measure of floating time in the FST was replaced with the measure of “time to sink” where a sink event is defined by the animal going entirely under water for approximately 2 seconds. Accordingly, right after the second sink, animals were taken out of the water by the experimenter and placed in their home cage and the test was terminated. Water in the cylinder was replaced after each test. Recordings were used to score the time of sink events by an experimenter blind to treatments.

### Heart weight

As previously described^37^, on week 22, the sand rats were euthanized, the chest cavity was rapidly opened, the heart removed and rinsed in two washes of ice-cold saline. Major blood vessels and connective tissue were removed, the heart blotted dry, weighed, and the heart weight/body weight ratio calculated.

### Statistical analysis

Statistical analysis was performed using STATISTICA 13.0 software (Dell, Tulsa, OK). Analysis of variance was utilized to explore statistical significance between diabetic (blood glucose levels that exceed 110 mg/dl) and non-diabetic animals. Initially we analyzed in one matrix one measure from each test, Sink 2 in the FST and time in the open arms in the EPM^54^. Following the overall analysis, we separately analyzed the results of the FST with a repeated measures ANOVA (with diabetic/not diabetic as main factor and Sink 1 and Sink 2 as repeated measure factor). One animal from the non-diabetic group was excluded as an outlier (more than 2XSTD away from the mean). The results from the EPM and heart weight ratio were analyzed with student’s t-test. Homogeneity of variance was analyzed using Levene’s test. Effect sizes were computed using Cohen’s d online calculator (https://www.uccs.edu/lbecker/) Correlations were performed using Pearson’s correlation test.
